# Eaten Out of House and Home: Impacts of Grazing on Ground-Dwelling Reptiles in Australian Grasslands and Grassy Woodlands

**DOI:** 10.1371/journal.pone.0105966

**Published:** 2014-12-11

**Authors:** Brett Howland, Dejan Stojanovic, Iain J. Gordon, Adrian D. Manning, Don Fletcher, David B. Lindenmayer

**Affiliations:** 1 Fenner School of Environment and Society, ANU College of Medicine, Biology & Environment Frank Fenner Building, # 141 Australian National University, Canberra, ACT, 0200, Australia; 2 James Hutton Institute, Invergowrie, Dundee, DD2 5DA, Scotland, United Kingdom; 3 Conservation Research, Environment and Sustainable Development Directorate, PO Box 158, Canberra, ACT, 2601, Australia; University of KwaZulu-Natal, South Africa

## Abstract

Large mammalian grazers can alter the biotic and abiotic features of their environment through their impacts on vegetation. Grazing at moderate intensity has been recommended for biodiversity conservation. Few studies, however, have empirically tested the benefits of moderate grazing intensity in systems dominated by native grazers. Here we investigated the relationship between (1) density of native eastern grey kangaroos, *Macropus giganteus*, and grass structure, and (2) grass structure and reptiles (i.e. abundance, richness, diversity and occurrence) across 18 grassland and grassy *Eucalyptus* woodland properties in south-eastern Australia. There was a strong negative relationship between kangaroo density and grass structure after controlling for tree canopy cover. We therefore used grass structure as a surrogate for grazing intensity. Changes in grazing intensity (i.e. grass structure) significantly affected reptile abundance, reptile species richness, reptile species diversity, and the occurrence of several ground-dwelling reptiles. Reptile abundance, species richness and diversity were highest where grazing intensity was low. Importantly, no species of reptile was more likely to occur at high grazing intensities. Legless lizards (*Delma impar*, *D. inornata*) were more likely to be detected in areas subject to moderate grazing intensity, whereas one species (*Hemiergis talbingoensis*) was less likely to be detected in areas subject to intense grazing and three species (*Menetia greyii*, *Morethia boulengeri*, and *Lampropholis delicata*) did not appear to be affected by grazing intensity. Our data indicate that to maximize reptile abundance, species richness, species diversity, and occurrence of several individual species of reptile, managers will need to subject different areas of the landscape to moderate and low grazing intensities and limit the occurrence and extent of high grazing.

## Introduction

Ecosystem engineers are species that alter the biotic and abiotic features of their environment and, by doing so, affect the availability of resources for other species in the habitats they occupy [1]. The means by which ecosystem engineers affect their environment vary widely, and include, among others, predation [2], deforestation [3], nutrient cycling [4], hydrological change [5] and herbivory [6]. Anthropogenic factors can affect how ecosystem engineers interact with their environment, with significant impacts on ecosystems [7]. For example, the human extermination of the gray wolf, *Canis lupus*, within Yellowstone National Park in the early 1900's resulted in long lasting and dramatic effects, including altered stream flow and localized extinction, that are only now being reversed with the reintroduction of the gray wolf [8]. The importance of ecosystem engineers in shaping the environment has made them the subject of intensive research [1], [9], [10] and management [11], [12]. Understanding the impact of anthropogenic change on ecosystem engineers is a priority for conservation because failure to do so can have dramatic, long lasting effects including loss of ecosystem function (e.g. introduced herbivores; [13]). In this study, we examine the effects of grazing by a native ecosystem engineer on native fauna in threatened grasslands and grassy woodlands across south-eastern Australia.

Large mammalian grazers (hereafter, grazers) are arguably among the most important ecosystem engineers in grassy habitats [14]. These ecosystems comprise a major proportion of the global landmass and biological diversity [15]. By trampling/eating vegetation and redistributing nutrients, grazers influence a range of key ecosystem functions and characteristics across multiple trophic levels [14], [16]. The intensity of their activity is one of the main drivers of how grazing affects the environment [14], [16]. For instance, intense grazing can increase mortality and reduce recruitment of plants leading to simplification of habitat structure and reduction in species diversity [14], [16], [17]. Conversely, suppression of grazing can allow a few plant species to competitively dominate, leading to a simplification of habitat structure and reduction in species diversity [14], [16], [17]. Consequently, grazing at moderate intensities is often recommended for biodiversity conservation (e.g. [18], [19]). However, there is currently limited empirical information on the benefits of moderate grazing intensities for biological conservation.

Anthropogenic impacts leading to a change in grazing intensity have occurred across terrestrial ecosystems globally [14], [16]. Several common themes have emerged for how anthropogenic changes affect grazing systems: (i) release from population suppression of grazers (e.g. predator removal) leading to increased grazing intensity, (ii) suppressed grazer populations (e.g. hunting) leading to reduced grazing intensity, and (iii) changes in the pattern of grazing (e.g. domestic livestock) leading to uniform grazing across the landscape [16], [18]. These changes can have profound consequences for ecosystem function [14], [16], [17]. Most grazing studies have focused on intense grazing by domesticated livestock in biologically depauperate production landscapes (e.g. [20], [21]). Relatively few studies have addressed the impact of changes in grazing intensity by native species in protected areas [22]. This gap in knowledge is important because protected areas are major reservoirs of biodiversity [23], and are often subject to anthropogenically altered grazing intensities [16]. Resumption of grazing patterns which promote conservation is a strategy adopted in restoration planning for many protected areas (e.g. Yellowstone National Park; [24]), but progress toward this goal is hampered by gaps in knowledge about what constitutes ‘appropriate’ grazing regimes for biodiversity conservation [16].

Ground-dwelling species are particularly vulnerable to changes in the intensity of grazing [25] and are an important component of biodiversity in grassy ecosystems [15]. Their vulnerability is due to their use of particular vegetation structures or configurations for food, shelter and reproduction [26]–[29]. Additionally, the relatively limited dispersal capacity of many ground-dwelling species prevents movement to better habitat when local conditions deteriorate [21], [30]. Ground-dwelling reptiles are particularly sensitive to grazing because, in addition to the above vulnerabilities, they are ectothermic and thus sensitive to microhabitat change [31]. Hence, reptiles are a good case study for investigating grazing impacts on ground-dwelling species [21]. Furthermore, relative to other taxa, the direct and indirect impacts of grazers on ground-dwelling reptiles are poorly understood [22]. Yet, these impacts may be profound [21], [29], [32]–[36]. Understanding these impacts is important because, in addition to their biodiversity value, reptiles provide an important ecological function in linking lower and upper trophic levels, as they largely feed upon invertebrates and plants, and are themselves then preyed upon by birds, mammals and other reptiles [37], [38].

In this study, we examined the impact of changes in grazing intensity by the native eastern grey kangaroo, *Macropus giganteus*, on reptiles in temperate grassland and grassy *Eucalyptus* woodland communities in south-eastern Australia. The eastern grey kangaroo (hereafter: kangaroo) is a medium-sized crepuscular and nocturnal marsupial (females; 40 kg, males; 90 kg) which occurs in open forests, woodland and grasslands, feeding predominately on grasses, with a minor component of browse [39]–[41]. Kangaroos are seen as ecosystem engineers in these environments [42], as their effect on the structure, and composition of grassy vegetation [41], [43]–[45] influences the resources available to other species [28], [46]. As a consequence of anthropogenic change, populations of kangaroos have been both inflated (e.g. by predator removal, establishment of artificial water points) and suppressed (e.g. by fencing, habitat fragmentation, hunting) across our study region [42]. This has resulted in a landscape-scale natural experiment characterized by a gradient of kangaroo densities and associated grazing intensities. We sampled reptiles along gradients in grazing intensity and asked: (1) Is there a relationship between the abundance of kangaroos and grass structure? (2) Does grazing at moderate intensities increase the abundance, richness, diversity and occurrence of reptiles? We aimed to provide baseline data on how changes in grazing intensity influence reptile communities, and provide recommendations for the management of grazing for conservation in these habitats.

## Methods and Materials

### Ethics statement

Reptile surveys were conducted with approval of the Australian National University Animal Experimentation Ethics Committee, protocol no. S.RE.10.10. There was a minor concern over impact on reptiles relating to tile searches, as turning of tiles may expose animals to predation. During surveys, care was taken to minimize adverse impact by returning tiles to initial position and not pursuing animals for identification for distances greater than 10 m. Ethical clearance was given for the handling of the threatened striped legless lizard, *Delma impar*. This was necessary to accurately separate records from the similar looking olive legless lizard, *Delma inornata*.

### Study region and Site selection

**Figure 1:**
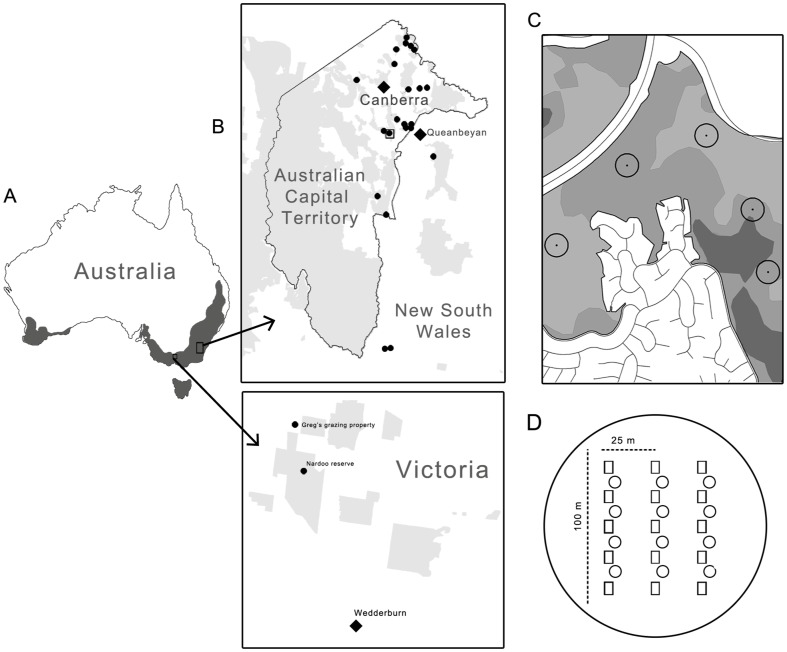
Study Design. a) The distribution of temperate grassland and grassy woodlands (grey) across Australia. Selected study regions are shown (black outline) and expanded in b) location of study sites within south-eastern NSW and central Victoria, with protected areas shown in grey, sites shown as black dots and major towns as large black diamonds, c) an example of stratified random placement of study plots within a study site showing open woodland (light grey), woodland (grey) and open forest (dark grey) canopy types, and d) the layout of tiles (box) and circular survey disks (circle) within the study plot.

Our study was conducted within the range of temperate grassland and grassy *Eucalyptus* woodland communities across south-eastern Australia (Fig. 1a). These threatened communities have been extensively cleared and modified over the past 200 years [47]. Remnant vegetation persists mostly as fragmented, often small (<1,000 ha) patches embedded in an agricultural matrix [47].

We selected 18 properties across the Australian Capital Territory (n = 14), New South Wales (n = 2) and Victoria (n = 2) where temperate grassland and grassy *Eucalyptus* woodland communities remain (Fig. 1b). The distribution of vegetation was closely aligned to topography and soil type; grasslands tend to occur in valley floors and clay soils, box-gum woodlands on lower slopes and fertile soils, with dry sclerophyll shrubby woodlands found on steeper slopes and infertile soils [48], [49]. Understorey was largely dominated by native perennial grasses (e.g. *Austrostipa spp., Bothriochloa macra, Rytidosperma spp., Themeda triandra*), although exotic perennial grasses were locally abundant at some locations (e.g. *Eragrostis curvula*, *Phalaris aquatica*). We assumed that by selecting sites where temperate grassland and grassy *Eucalyptus* woodland communities remained, we sampled sites with a relatively intact reptile community, as the reasons for decline of these communities (i.e. disturbance) have also negatively impacted reptiles [33], [50]. To avoid issues of small habitat islands [51], we selected large properties (>100 ha), that were, or were until recently (<15 years), connected to much larger (>500 ha) patches of remnant vegetation. Thus, we consider it unlikely that fragmentation influenced differences in fauna composition between properties [51]. At the time of study, all but one property was being managed for the conservation of biodiversity, with a single property managed for livestock production. At this property, livestock had not been present for at least 12 months prior to study commencement. While selected properties covered a large geographical area, many of the same reptile species potentially occurred across all properties [37], [38]. We did not consider geographic differences, as our intention was to explore whether the effects of changes in grass structure on reptiles was consistent across wide geographical areas. All sites fell within the ‘temperate cool-season wet’ climatic pattern with rainfall fairly evenly distributed throughout the year [52]. The impacts of grazing on vegetation have likely been exacerbated by drought conditions which have prevailed over the study region for the last decade [53].

The kangaroo is the most common large native herbivore encountered in south-eastern Australia, and is considered to have benefited from European land-use change and eradication of the largest remaining carnivore, the dingo, *Canis lupus dingo* (see [39]). Population surveys using pellet counts in the ACT and NSW, have confirmed the relative dominance of kangaroos compared to other large herbivores [42]. Historically, the European rabbit, *Oryctolagus cuniculus*, has been a major contributor to grazing pressure; however national control programs (i.e. disease release; [54]) and ongoing management at all selected properties appear to have controlled rabbit numbers. Under current management, the contribution of rabbit grazing to total grazing pressure, appears to be substantially less than that of kangaroos [55]. Both species of grey kangaroo, the eastern grey kangaroo and the western grey kangaroo, *Macropus fuliginosus*, were sympatric at Victorian properties, although the former was more common. These species are known to interbreed, and occupy similar niches, although western grey kangaroos appear more adapted to arid environments [39], [56]. For the purposes of this study, we consider the impacts of these two species on grass to be comparable.

While all sites had a legacy of fire and grazing by domesticated livestock, with the exception of three properties, all properties had not been grazed by livestock or burnt in the last 10 years and no properties had been burnt or grazed by livestock within a year prior to study. We consider grazing by kangaroos to be the greatest contributor to grazing pressure.

We selected properties to cover a range of grazing intensities (i.e. low, medium, high) on the basis of initial visual observations of grass attributes. These categories were not used in analysis, but instead ensured we sampled from a range of grazing intensities. In most cases, the level of grazing was considered to be similar across an entire property. However, on a few properties barriers to movement (fencing, topography) had resulted in different levels of grazing being experienced. We took advantage of these differences, by stratifying sampling effort between these gradients. We recognised 24 different grazing units (hereafter: sites) across our 18 properties (see Appendix S1). A license to undertake this research on ACT reserves was obtained from the Land Management and Planning Division of the ACT Government prior to project commencement. Additionally, permission to access Bush Heritage, Department of Defence, and a single private property were obtained from relevant authorities.

### Experimental Design

Tree canopy cover influences the distribution of reptiles [57], grass productivity [58] and large herbivore grazing patterns [59]. We therefore stratified our sites by canopy cover (Fig. 1c). A tree canopy cover map was created for each site using an unsupervised ISODATA classification technique [60] in Multispec [61] based on aerial photographs taken in 2008/9. The resulting map was further delineated into: grassland (0–2% canopy cover), open woodland (2–20%), woodland (20–50%), open forest (50–80%) and forest (80–100%) canopy class polygons (>1 ha). These categories were similar to those in the national vegetation information system [62].

Within each site, we established multiple reptile survey plots (hereafter: plots) using a stratified random design, within grassland, open woodland and woodland canopy classes. We did not sample open forest or forest canopy classes as the grassy layer is suppressed in these dense habitat types. Two grassland plots were reclassified as open woodland due to the presence of several small trees within plot boundaries. Between three and nine 75 m radius (1.8 ha) plots were established at each site, with more plots established at larger sites with a greater variety of tree canopy classes (Fig. 1c). We used plots of this size because they are known to be effective in studies of reptile-habitat relationships [21]. Overall, 127 plots were established, with 36 plots in grassland, 50 in open woodland, and 41 in woodland canopy classes.

### Habitat characteristics

Tree canopy cover is correlated with important habitat features for reptiles (e.g. leaf litter and woody debris) and may directly affect habitat quality for reptiles by reducing light penetration [63]. We accounted for the potential confounding effect of tree canopy cover on reptiles by including tree canopy cover class as a dummy variable in analysis.

### Grazing intensity

We used several grass attributes (see below) as a surrogate for kangaroo grazing intensity, rather than direct measures of grazing intensity (i.e. herbivore abundance). This approach had several advantages. First, we measured the resource for which reptiles interact directly for food and shelter (i.e. grass). Second, estimates of herbivore abundance are more error-prone than measures of vegetation condition [64], [65]. Third, grass structure provided an integrated, quantitative index of grazing impact among sites that vary in grazing history, soil and rainfall [16], [66]. The value of grass structure as a surrogate for grazing pressure was tested by relating grass attributes to kangaroo abundance at a select number of sites where conditions were relatively similar.

### Grass attributes

We conducted grass surveys from December 2009 to February in 2010 (coinciding with the local peak in perennial grass growth). We surveyed grass layer attributes within twelve 0.25 m^2^ circular survey disks, which were systematically placed across each plot (Fig. 1d). At each grass survey point, we recorded grass height (cm), biomass (kg dry matter/ha), and percent cover of grass. We estimated grass height as the average height of above-ground leaf, with the ‘comparative yield’ technique [67] used to estimate grass biomass. We took vertically-oriented photographs of each grass survey point and analyzed them using the program ‘SamplePoint’ [68] to calculate percentage grass cover. Averaged over the plot grass height ranged from 1.7–18.1 cm, grass biomass from 66–3354 kgDM/ha and percent grass cover from 0–91%.

### Kangaroo density

We estimated the density of kangaroos within different tree canopy classes at a select number of sites (n = 15). We restricted analysis to sites where conditions were relatively similar to avoid factors like grazing history and rainfall confounding the relationship between herbivore abundance and grass attributes [16]. A variety of established methods were employed to survey kangaroos - pellet counts, total counts and line transect distance sampling (see [65]). Details of kangaroo surveys can be found in Appendix S2. We believe these surveys of kangaroo abundance provided a representative assessment of grazing pressure within different tree canopy classes. For example, because kangaroo defecate more while feeding [69], pellet counts provide an unbiased assessment of grazing pressure. Total counts were employed only at sites where a single canopy class occurred. These sites were small and it was possible to count the entire population from vantage points. Counts were repeated on multiple days to ensure we captured an accurate representation of grazing pressure. Line transect distance sampling was undertaken early in the morning when kangaroos are actively feeding to provide an account of grazing pressure between canopy classes. We estimated kangaroo density in 2009 at 12 sites, and in 2011 at an additional three sites. Kangaroo management activities were undertaken at a single study site in the winter of 2009. Therefore, we estimated density for this site as the average between the 2008 (pre management) and 2009 (post management) kangaroo density estimates. The density of kangaroos ranged from 0.25 to 3.6 animals/ha across selected sites.

### Reptile surveys

In 2010 we surveyed plots for ground-dwelling reptiles using artificial shelters (as per [70]). We conducted reptile sampling from February to May and from September to November, as reptiles are more likely to use artificial shelters during this time [70]. We deployed artificial shelters comprising 15 concrete roof tiles at each plot 2–3 months before reptile surveys. We did this to increase capture rates by allowing time for reptiles to grow accustomed to (and then occupy) the tiles. We spaced tiles at least 15 m apart (Fig. 1d), which is larger than the home range and movement of many small reptiles [71]. This reduced the likelihood of double counting, which was important as we considered tiles as independent sampling units for our estimation of abundance.

We conducted searches of tiles for basking and sheltering reptiles on non-rainy days, after sunrise and before sunset during periods of mild temperature (i.e. 5–25°C). To increase the likelihood of presence and reduce bias occurring due to variation in weather conditions, we surveyed each tile and plot on eight separate occasions. We assumed that habitat structure did not affect the likelihood of a reptile being detected under a tile. Rather, tiles provided a temporary snapshot of reptile abundance in surrounding habitat. This assumption appeared to be valid as the thermal properties of tiles (temperature extremes) prevented their permanent occupation. We could not complete reptile searches at two plots due to difficult terrain and damage to tiles, leaving a total of 125 plots for analysis.

### Analysis

We performed a principal components analysis (PCA) using a correlation matrix on all grass attributes (grass height, grass biomass, grass cover) to reduce the number of covariates, and obtain a smaller number of independent variables [72]. PCA values are the combination of multiple component variables, and cannot be quantified directly in the field, making interpretation by managers problematic. Therefore, to aid interpretation of results, we examined the relationship between each component variable and PCA values, using generalized linear modeling procedures (GLM; [73]). We modeled this relationship with a normal distribution and an identity link function.

Next we explored the relationship between PCA values, kangaroo density and tree canopy class using hierarchical generalized linear mixed modeling procedures (HGLMM; [74]). Due to the non-trivial issues of model selection where random effects are included [75], we opted to fit a complete model for each analysis, instead of undertaking the more common approach of choosing a ‘best’ model on the basis of AIC values (i.e. [76]). We included tree canopy cover class, as canopy cover can reduce grass growth [58]. We estimated kangaroo abundance over an entire canopy class, because the size of individual plots was at too fine a scale to estimate kangaroo abundance accurately [65]. To obtain values for each canopy class, we averaged PCA for all plots within the same canopy class. To account for nesting and potential similarity between canopy classes within the same property, we included property as a random effect [74]. The relationship was modeled with a normal distribution and an identity link function, with random effects fitted with a normal distribution and an identity link function. Prior to regression analysis, we transformed PCA values (variable +1.5) to obtain positive values.

We calculated reptile abundance, species richness, and species diversity for each plot. We defined reptile abundance as the maximum number of individuals of a species seen at the tile in a single visit. We then summed values for all species to estimate abundance at the plot level. We defined species richness as the total number of species recorded for each plot after eight visits. We calculated diversity using the Shannon index, as this index appeared appropriate given the numerical dominance of a few species in our results [77].

We built occupancy models for those species of reptiles which were encountered in >10% of plots, namely: Boulenger's skink, *Morethia boulengeri* (46%), delicate skink, *Lampropholis delicata* (41%), common dwarf skink, *Menetia greyii* (23%) and the eastern three-toed earless skink, *Hemiergis talbingoensis* (16%). The striped legless lizard, *Delma impar* (9%) and the olive legless lizard, *Delma inornata*, (6%) had small sample sizes but have similar life histories [37], [78], so were pooled in a ‘legless lizard’ group (14%) for the analysis. The delicate skink is not known to occur near our Victorian properties [38]. We therefore removed all plots in Victoria (n = 9) from analysis for this species. For analysis of reptile habitat relationships we reclassified two grassland plots as open woodland, due to several small trees occurred within plot boundary. To account for zero-dominated occupancy data, we analyzed reptile data as presence/absence in a logistic regression. We considered a reptile to be present at a tile if it was recorded at least once in any of the eight visits.

Lastly, we modeled reptile abundance, species richness, species diversity and individual reptile occupancy as a function of PCA values and tree canopy cover class using HGLMM procedures. To account for nesting (i.e. plots occurred within a property) and potential similarity between plots within our study design, we included property as a random effect [74]. Again, we fit a complete model, with grass structure and tree canopy class included. The relationship between reptile abundance and species richness was modeled with a Poisson distribution, a logarithm link function, and random effects fitted with a gamma distribution and a logarithm link function. Reptile diversity was modeled with a normal distribution and an identity link function, with random effects fitted with a normal distribution and an identity link function. We modeled individual species with a binomial distribution, a logit link function, with random effects fitted with a beta distribution and a logit link function. Grass structure was fitted as a linear term, with a quadratic term included where exploratory analysis indicated a possible curvilinear relationship. Residual plots and distribution of residuals were examined to check model assumptions. We standardized grass structure to a mean of 0 and standard deviation of 1. All analyses was undertaken in Genstat 12 [79].

## Results

We recorded 781 reptiles, representing 20 species from four families, including 89 records of ‘unidentified skinks’ (Appendix S3).

### Principal components analysis

We found the first two components of the PCA described 95.15% of the variation between grass attribute variables. The first PC explained 81.35% of variation with a latent root value of 2.441. High values corresponded to tall grass, large amounts of biomass and high grass cover with low values corresponding to short grass, low amounts of biomass and limited cover of grass. Principal component one collapses grass metrics into one unit which here we call ‘grass structure’. The second PC explained only 13.8% of variation with a latent root value 0.414. We dropped the second PC as factors with latent roots less than one are considered insignificant [80]. We found a significant positive relationship between the first PCA (i.e. grass structure), and each component grass attribute (Appendix S4). To aid interpretation, we graphically represented the relationship between grass structure and each component grass attribute in Appendix S5.

### Grazing intensity

**Figure 2:**
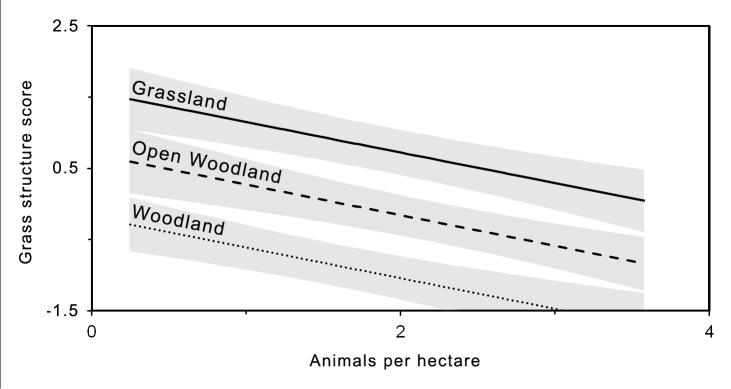
Graphical representation of linear regression models summarized in Table 1. The graph shows significant relationships between kangaroo density (animals/ha) and grass structure (PC1) for each tree canopy cover class (grassland, open woodland, woodland). Standard error of the model predictions are shown in grey. To aid interpretation of results, variables have been back-transformed to original scale.

Grass structure was significantly and negatively related to both tree canopy cover and kangaroo density (Table 1). Our results suggest, that to maintain the same level of grass structure, more kangaroos could be supported in grasslands, with low tree canopy cover, as compared to open woodlands and woodlands, where tree canopy cover is high (Fig. 2).

**Table 1:** Results of hierarchical generalized linear mixed models of PC1 (grass structure) in relation to kangaroo density and tree canopy class (dummy variable) showing trends (Slope) including standard errors (SE).

Response	Model term	d.f.	?2	Slope	SE	Graphical summary
Grass structure	intercept			3.08	0.45	
	tree canopy class	2	27.81***			Fig. 2
	open woodland			−0.88	0.32	
	woodland			−1.76	0.34	
	kangaroo abundance	1	5.94*	−0.43	0.21	Fig. 2

Grassland has been used as a reference level for tree canopy class categories. Significance is indicated by the Wald statistic (χ2) and p-value as follows: *p<0.05, ** p<0.01, ***p<0.001.

### Habitat relationships

**Figure 3:**
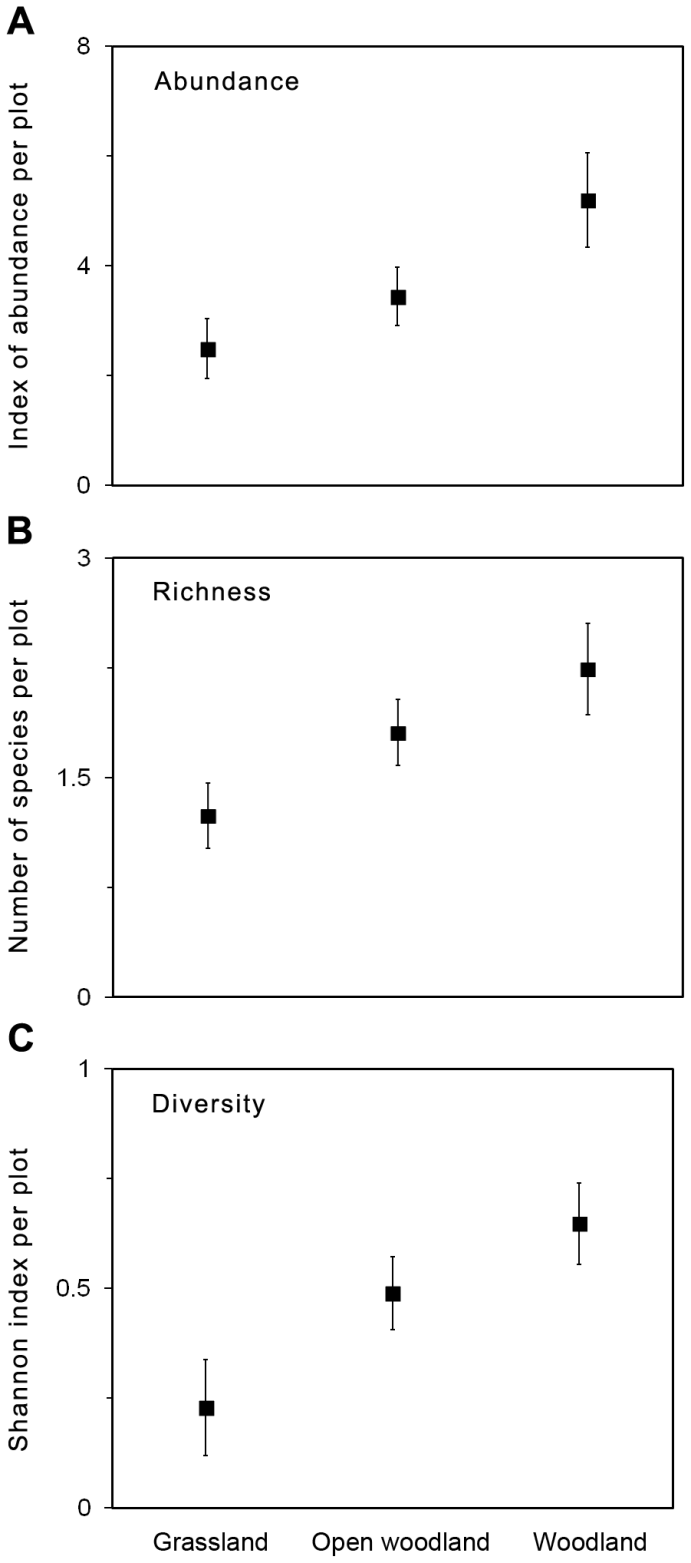
Graphical representation of logistic regression models summarized in Table 2. The graphs shows mean values for a) reptile abundance, b) reptile species richness and c) reptile species diversity at a given plot for each tree canopy cover class (grassland, open woodland, woodland). Standard error bars are shown. To aid interpretation of results, variables have been back-transformed to original scale.

**Figure 4:**
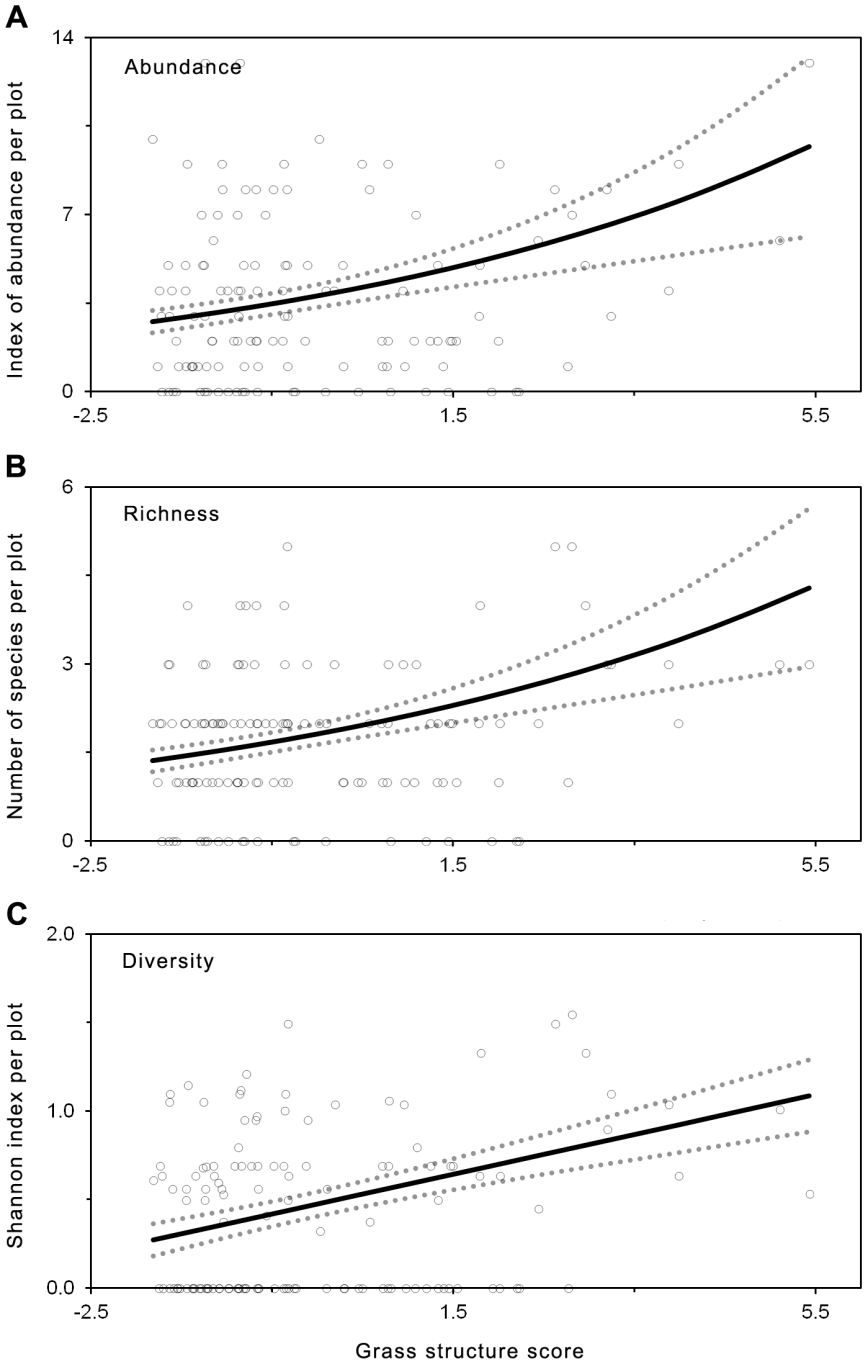
Graphical representation of linear regression models summarized in Table 2. The graphs show significant relationship between grass structure (PC1) for a) reptile abundance, b) reptile species richness and c) reptile species diversity in a given plot. Fitted relationships are shown as a solid line, with actual values shown as an open circle. Standard error of the model predictions are shown as a dotted line. To aid interpretation of results, variables have been back-transformed to original scale.

We developed HGLMM for reptile abundance, species richness, species diversity, and the occurrence of Boulenger's skink, delicate skink, common dwarf skink, eastern three-toed earless skink, and legless lizards (Table 2). Tree canopy cover was an important predictor of reptile abundance, species richness and diversity, with highest values recorded in woodland habitat (Fig. 3). Reptile abundance, species richness and diversity were positively related to grass structure (Fig. 4).

**Table 2:** Results of hierarchical generalized linear mixed models for species abundance, species richness, reptile diversity, and individual species showing trends (Slope) including standard errors (SE).

Response	Model term	d.f.	?^2^	Slope	SE	Graphical summary
Reptile Abundance	Intercept			0.91	0.22	
	tree canopy class	2	8.006*			Fig. 3a
	open woodland			0.32	0.24	
	woodland			0.74	0.28	
	grass structure	1	7.589**	0.27	0.10	Fig. 4a
Species Richness	Intercept			0.21	0.18	
	tree canopy cover	2	6.511*			Fig. 3b
	open woodland			0.38	0.20	
	woodland			0.59	0.23	
	grass structure	1	8.627**	0.24	0.08	Fig. 4b
Reptile Diversity (Shannon index)	Intercept			0.23	0.11	
	tree canopy class	2	9.74**			Fig. 3c
	open woodland			0.26	0.12	
	woodland			0.42	0.13	
	grass structure	1	10.49**	0.17	0.05	Fig. 4c
Boulenger's skink	Intercept			−4.27	0.56	
	tree canopy class	2	10.461**			Fig. 5a
	open woodland			1.72	0.59	
	woodland			2.06	0.64	
	grass structure	1	0.017	−0.03	0.22	
Delicate skink	Intercept			−3.68	0.41	
	tree canopy class	2	20.33			Fig. 6b
	open woodland			0.39	0.43	
	woodland			1.69	0.48	
	grass structure	1	3.33	0.28	0.15	
Common dwarf skink	Intercept			−4.24	0.53	
	tree canopy class	2	1.917			
	open woodland			0.69	0.57	
	woodland			0.44	0.67	
	grass structure	1	1.504	0.37	0.31	
	grass structure∧2	1	2.507	−0.37	0.23	
Eastern three-toe earless skink	Intercept			−5.61	0.54	
	tree canopy class	2	6.3*			Fig. 5c
	open woodland			−0.12	0.37	
	woodland			1.07	0.56	
	grass structure	1	35.26***	1.15	0.19	Fig. 6a
Legless lizard	Intercept			−4.34	0.61	
	tree canopy class	2	7.09*			Fig. 5d
	open woodland			−0.89	0.34	
	woodland			−10.00	16.00	
	grass structure	1	14.77***	2.44	0.63	Fig. 6b
	grass structurê2	1	14.17***	−0.89	0.24	

Grassland has been used as a reference level for tree canopy class categories. Significance is indicated by the Wald statistic (χ2) and p-value as follows: *p<0.05, ** p<0.01, ***p<0.001.

**Figure 5:**
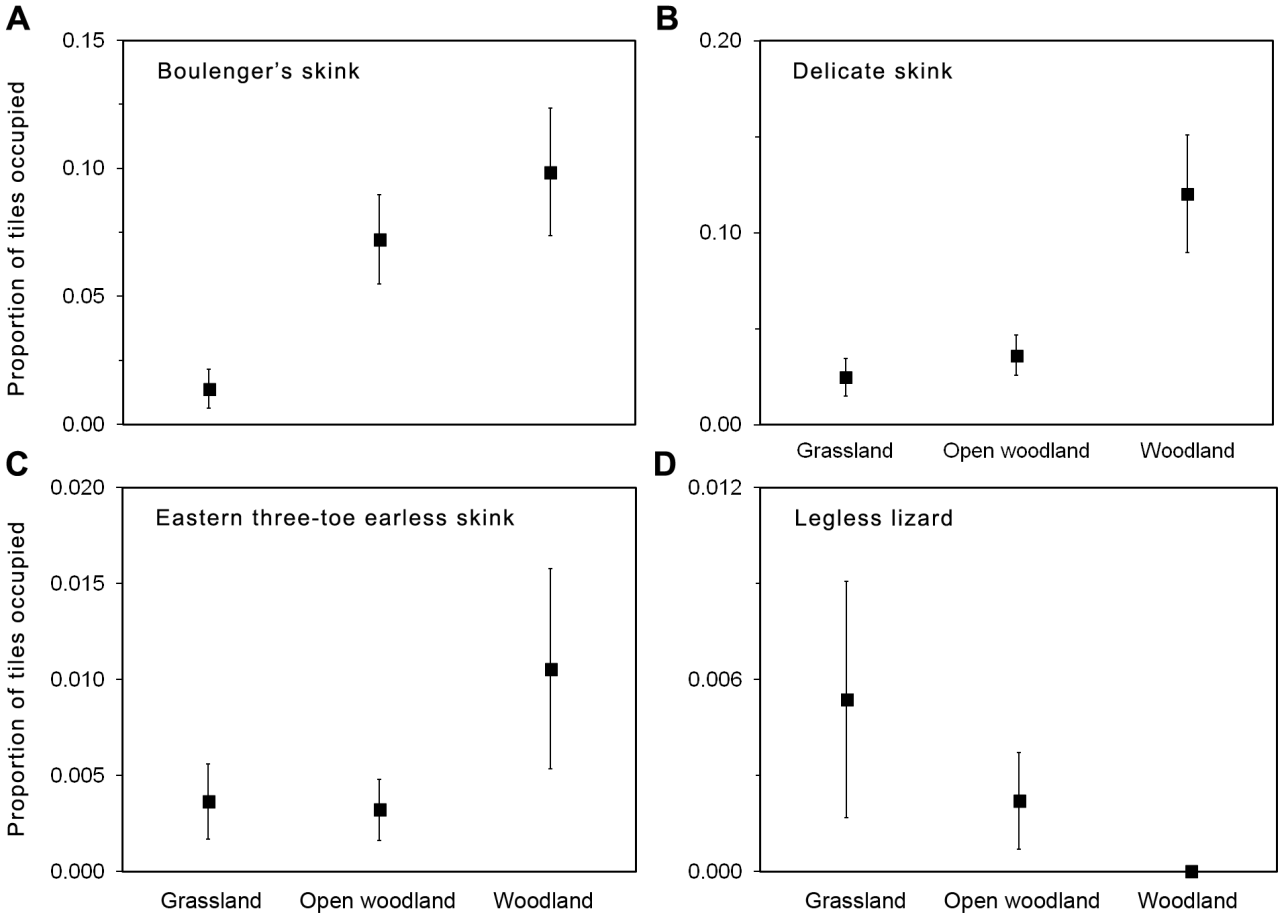
Graphical representation of logistic regression models summarized in Table 2. The graphs shows mean values for a) Boulenger's skink, b) delicate skink, c) eastern three-toe earless skink and d) legless lizard at a given plot for each tree canopy cover class (grassland, open woodland, woodland). Standard error bars are shown. To aid interpretation of results, variables have been back-transformed to original scale.

**Figure 6:**
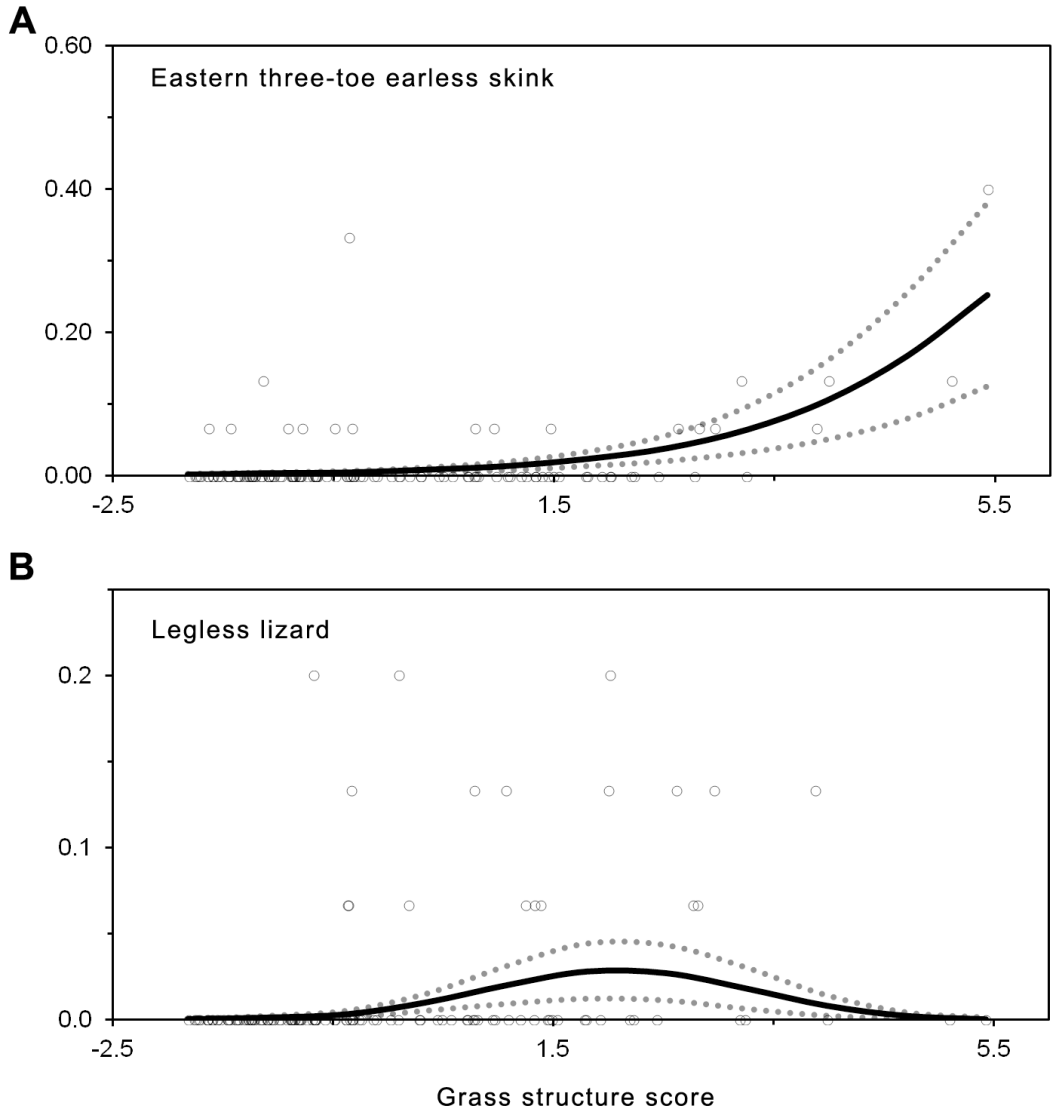
Graphical representation of logistic regression models summarized in Table 2. The graphs show significant relationships between PC1 values (grass structure) and the probability of encountering a species in a given plot for the a) eastern three-toe earless skink, and b) legless lizard. Fitted relationships are shown as a solid line, with actual values shown as an open circle. Standard error of the model predictions are shown as a dotted line. To aid interpretation of results, variables have been back-transformed to original scale.

Boulenger's skink and the delicate skink did not respond to grass structure but responded significantly to changes in tree canopy cover. The occurrence of the Boulenger's skink, delicate skink and the eastern three-toed earless skink increased with increasing tree canopy cover, with all three species most common under woodland habitat (Fig 5). In contrast, legless lizard presence was significantly negatively related to tree canopy cover, and was most common under grassland habitat, and did not occur under woodland habitat (Fig. 5d). The likelihood of occurrence of the eastern three-toed earless skink, and legless lizard, were significantly related to grass structure (Fig. 6). The occurrence of the eastern three-toed earless skink increased with increasing values of grass structure (Fig. 6a). The legless lizards were more likely to occur at intermediate grass structure values between 1–3 (Fig. 6b). Occurrence of the common dwarf skink was not related to any environmental variable.

## Discussion

We have shown that the abundance, species richness, species diversity and occurrence of ground-dwelling reptiles varied over a natural gradient in grazing intensity related to the density of a native grazer, the eastern grey kangaroo. Crucially, a strong and inverse relationship between kangaroo density and grass structure existed, allowing us to use grass structure (i.e. PC1) as a surrogate for grazing intensity. Our study revealed that changes in grazing intensity affected different species in different ways. Importantly, intense grazing and associated reduction in grass structure was not favoured by any reptiles in this study. We found reptile abundance, reptile species richness, reptile diversity and the occurrence of three of six species of reptile were all lower at high grazing intensity. We found only limited evidence to support the benefits of grazing at moderate intensities in these landscapes. Therefore, across the landscape, managers will need to subject areas to different grazing intensities, to accommodate varied requirements of reptiles. We suggest that even in habitats dominated by native grazers, changes in grazing intensity can have major impacts on ground-dwelling species.

### General impacts of grazing

Changes in grazing intensity by domestic livestock have been shown to have a profound effect on the reptile community. Generally, intense grazing by domestic livestock has had a negative effect on both reptile abundance and reptile species richness [29], [32]–[36], [81]. However, studies by Brown et al. [21] and Dorrough at al. [57] in Australian grassy woodlands, found a weak but positive effect of the number of domestic herbivores on reptile abundance. This positive effect of grazing appeared to be driven by an increase in abundance of a few generalist species that benefit from reduction in ground cover with increased grazing [21], [57]. In this study, reptile species diversity was positively correlated with grazing intensity, with low grass structures also dominated by just a few generalist species. However, at high grazing intensities, the reptile community was less diverse, reptiles were less common and reptile species richness was lower. The only other study on native grazers and reptiles in grassy woodlands in Australia found the experimental reduction in grazer numbers resulted in increased small skink abundance [46]. Prolonged intense grazing resulting in the simplification of grass structure may negatively impact reptiles through increased predation risk [35], decreased prey availability [28], and loss of shelter [32], [35]. Importantly, the impacts of grazing on reptiles is likely to cascade up the food chain as reptiles are important food sources for various other taxa, including birds, mammals and large predatory reptiles [38].

Habitat features associated with tree canopy cover, such as leaf litter and logs are known to provide important habitat features for reptiles [32], [78]. In this study, reptile abundance, reptile diversity and reptile species richness was positively associated with tree canopy cover, and was higher in woodland than grassland habitats (Fig. 3). Utilization of non-grass structures may buffer the impacts of grazing by providing alternate habitat refugia (e.g. [28], [46]). The implications for land managers of this result is that the management of grazing can be critical in grasslands which lack tree-related habitat refugia.

In addition to generating new information on broad interactions between grazing intensity and reptiles, our study provides new insights into the habitat preferences of several poorly researched reptiles. For example, legless lizards were most common in areas with moderate grazing intensity (Fig. 6b), and low tree canopy cover (Fig. 5d). While preference for low tree canopy cover is well established [21], [78], our study is the first study to identify preference for a particular grazing intensity. The eastern three-toed earless skink was more likely to occur in areas subject to under low grazing intensity (Fig. 6a), and was more common in woodland compared to open woodland and grassland habitats (Fig. 5c). The vulnerability of the eastern three-toe earless skink to intense grazing has not previously been recorded.

Unlike the other species, Boulenger's and delicate skinks did not respond to grass attributes, but instead were positively associated with tree canopy cover (Fig. 5a and 5b). The preference of these species for leaf litter and logs [82], which are not directly impacted by grazing, may account for this result. Nonetheless, high intensity grazing may still impact these species over longer temporal scales via reduced tree recruitment [78], or by impacting invertebrate prey [28].

### Management prescriptions

Managers require optimal grazing conditions to be expressed as recommended animal densities under a range of conditions [16]. The modeled relationship between kangaroo abundance and grass structure (Fig. 2) in this study could be used to develop such recommended herbivore densities based on optimal grass structure for reptiles. For instance, based on optimal grass structure for legless lizards (Fig. 6b), our model predicts a kangaroo density less than 0.5 kangaroos per hectare. However, this relationship is based on average values, at a select number of sites over a few years, thus the relationship will unlikely hold in different sites and different years. Instead of defining management prescriptions, the association between kangaroo abundance and grass structure has several important implications for managers. First, grass structure had a negative relationship to the number of kangaroos. Second, the impact of kangaroo grazing was greater where canopy cover was higher (i.e. open woodlands and woodlands). Third, regardless of kangaroo densities, grass structure was low in woodland habitat. To obtain more detailed recommendations for animal density under a range of conditions, a greater understanding of factors that affect grazing impact (e.g. grass growth and herbivore consumption) will be required.

### Conservation implications

Grazing at moderate levels is often recommended for biodiversity conservation (e.g. [17], [18], [83]), as this level of grazing is considered to increase niche availability, and hence species richness [84]. In our study, although legless lizards (olive legless lizard, striped legless lizard) preferred moderate grazing intensity, the abundance, richness and diversity of reptiles and occurrence of the eastern three-toe skink was highest at low grazing intensity. Hence, moderate grazing intensity did not ensure conservation of all ground-dwelling reptiles in this study. In reality, few empirical studies have found support for grazing at moderate intensities for biological conservation (see [57], [85], [86]) as different species require different habitat conditions. The challenge for grazing management in these environments is highlighted in this study by the preference of the threatened striped legless lizard [87] for moderate grazing intensity, rather than low grazing intensities which were associated with the most diverse reptile assemblage. Therefore, to accommodate varied requirements of reptiles, there will be a need for land managers to subject different areas of the landscape to different grazing intensities [18], [34], [88].

Our results indicate that to maximize habitat quality simultaneously for multiple species and increase reptile abundance, diversity and richness, prolonged intense grazing resulting in areas with low grass structure should be avoided. However, a common consequence of anthropogenic management in protected areas in Australia (and elsewhere) has been an increase in herbivore density and grazing pressure [16], [89]. For reptile species like those in our study that prefer light to moderate grazing intensities, inflation of grazer populations can profoundly alter habitat suitability. The sensitivity of reptiles in our study is likely to be symptomatic of broader problems facing a range of species in habitats shaped by grazing. Anthropogenically altered patterns and intensity of grazing has repeatedly been shown to negatively impact biodiversity [14], [16], [22], and our results conform to these broad trends for a native species of grazer. Evidence from more intensively researched study systems under livestock grazing in Australia has shown that management approaches that support both low to moderate grazing intensity across the landscape, and that limit prolonged intensive grazing, are likely to benefit biodiversity [29], [85], [90], [91]. Limiting prolonged intense grazing in systems dominated by native herbivores typically relies on the re-introduction of predators [8], and/or culling [12].

## Conclusions

Our study provides important new insights into the impact of native herbivore grazing pressure, and indicates that intense grazing does not support an abundant or rich reptile community. In line with other studies on grazing sensitive systems around the world, the recognition of certain grazers as important ecosystem engineers, and their management in anthropogenically altered landscapes is important for the maintenance of biodiversity in grassy habitats.

## Supporting Information

Appendix S1 **Characteristics of reptile study sites** **(listed in alphabetical order).**
(DOC)

Appendix S2 **Details of the study site, canopy class, density obtained, method used and year kangaroo surveys were carried out at 14 study sites.**
(DOC)

Appendix S3 **Reptiles captured in this study, ordered by “Number of records”.**
(DOC)

Appendix S4 **Results of generalized linear mixed models for grass biomass, grass height and grass cover showing trends (Slope) including standard errors (SE).**
(DOC)

Appendix S5 **Graphical representation of generalized linear regression model summarized in Appendix S4.**
(DOC)
