# Supplementary material for: Eaten Out of House and Home: Impacts of Grazing on Ground-Dwelling Reptiles in Australian Grasslands and Grassy Woodlands
Source: PLoS One. 2014 Dec 11;9(12):e105966. doi: 10.1371/journal.pone.0105966 (PMC4263405; doi:10.1371/journal.pone.0105966)
Supplement: Appendix S1 — Characteristics of reptile study sites (listed in alphabetical order). (DOC) [file pone.0105966.s001.doc]

Appendix S1: Characteristics of reptile study sites (listed in alphabetical order). “Governance” is the state or territory in which study site was located, ACT= Australian Capital Territory, NSW = New South Wales, VIC=Victoria. “Estimated grazing intensity”, grazing category based on visual inspection of grass attributes.

| Property ID | Grazing unit ID | Governance | Estimated Grazing intensity | Area - Property | Area - Grazing unit | Number of canopy types | Number of reptile Plots |
| --- | --- | --- | --- | --- | --- | --- | --- |
| 1 | 1 | ACT | Low | 106 | 106 | 2 | 4 |
| 2 | 2 | ACT | High | 143 | 143 | 2 | 6 |
| 3 | 3 | ACT | High | 112* | 13 | 1 | 3 |
| 4 | 4 | ACT | Low | 113 | 113 | 1 | 3 |
| 7 | 7 | ACT | Moderate | 179 | 179 | 2 | 4 |
| 8 | 6 | ACT | Moderate | 239 | 239 | 2 | 6 |
| 9 | 7 | NSW | High | 530 | 530 | 3 | 9 |
| 6 | 8 | ACT | Moderate | 688 | 112 | 2 | 4 |
| 10 | 8 | ACT | Moderate | 688 | 576 | 2 | 7 |
| 12 | 9 | ACT | Low | 112 | 15 | 1 | 3 |
| 11 | 9 | ACT | Moderate | 112 | 97 | 1 | 3 |
| 13 | 10 | ACT | Low | 240 | 15 | 1 | 3 |
| 14 | 10 | ACT | Moderate | 240 | 225 | 3 | 7 |
| 15 | 11 | ACT | Low | 154 | 154 | 3 | 9 |
| 16 | 12 | ACT | High | 701 | 581 | 3 | 8 |
| 17 | 12 | ACT | Low | 701 | 120 | 1 | 3 |
| 18 | 13 | ACT | Low | 142 | 142 | 2 | 6 |
| 4 | 14 | ACT | Moderate | 429 | 77 | 2 | 6 |
| 19 | 14 | ACT | High | 429 | 352 | 2 | 6 |
| 24 | 18 | VIC | High | 825 | 825 | 2 | 6 |
| 23 | 17 | VIC | High | 221 | 221 | 2 | 3 |
| 21 | 15 | NSW | Moderate | 1151 | 790 | 2 | 6 |
| 20 | 15 | NSW | Low | 1151 | 361 | 2 | 6 |
| 22 | 16 | ACT | High | 229 | 229 | 2 | 6 |

* Approximation
