# Supplementary material for: Eaten Out of House and Home: Impacts of Grazing on Ground-Dwelling Reptiles in Australian Grasslands and Grassy Woodlands
Source: PLoS One. 2014 Dec 11;9(12):e105966. doi: 10.1371/journal.pone.0105966 (PMC4263405; doi:10.1371/journal.pone.0105966)
Supplement: Appendix S2 — Details of the study site, canopy class, density obtained, method used and year kangaroo surveys were carried out at 14 study sites. (DOC) [file pone.0105966.s002.doc]

Appendix S2: Details of the study site, canopy class, density obtained, method used and year kangaroo surveys were carried out at 15 study sites. “G”, grassland, “OW”, open woodland, “W”, woodland.

| Grazing unit - ID | Tree canopy class | Density | Method | Year |
| --- | --- | --- | --- | --- |
| 1 | OW | 0.6 | Pellet count | 2011 |
| 1 | W | 2 | Pellet count | 2011 |
| 2 | OW | 2.2 | Pellet count | 2009 |
| 2 | W | 1.6 | Pellet count | 2009 |
| 3 | G | 3 | Total count | 2009 |
| 4 | G | 1.2 | Total count | 2009 |
| 7 | OW | 3.6 | Pellet count | 2011 |
| 7 | W | 1.4 | Pellet count | 2011 |
| 6 | G | 1.2 | Pellet count | 2009 |
| 6 | OW | 1.5 | Pellet count | 2009 |
| 8 | OW | 1.5 | Pellet count | 2011 |
| 8 | W | 1.8 | Pellet count | 2011 |
| 9 | G | 2.7 | Pellet count | 2009 |
| 9 | OW | 2.1 | Pellet count | 2009 |
| 9 | W | 1.7 | Pellet count | 2009 |
| 10 | OW | 2.7 | Pellet count | 2009 |
| 10 | W | 1.5 | Pellet count | 2009 |
| 11 | G | 3 | Total count | 2009 |
| 14 | G | 0.5 | Pellet count | 2009 |
| 14 | OW | 2.4 | Pellet count | 2009 |
| 14 | W | 1.9 | Pellet count | 2009 |
| 18 | G | 0.7 | Pellet count | 2009 |
| 18 | OW | 1.5 | Pellet count | 2009 |
| 19 | OW | 2 | Pellet count | 2009 |
| 19 | W | 1.6 | Pellet count | 2009 |
| 22 | OW | 2.3 | Pellet count | 2009 |
| 22 | W | 0.2 | Pellet count | 2009 |
| 16 | G | 3.4 | Distance sampling | 2008/2009 |
| 16 | OW | 2.8 | Distance sampling | 2008/2009 |
| 16 | W | 1.7 | Distance sampling | 2008/2009 |
