# Supplementary material for: Eaten Out of House and Home: Impacts of Grazing on Ground-Dwelling Reptiles in Australian Grasslands and Grassy Woodlands
Source: PLoS One. 2014 Dec 11;9(12):e105966. doi: 10.1371/journal.pone.0105966 (PMC4263405; doi:10.1371/journal.pone.0105966)
Supplement: Appendix S3 — Reptiles captured in this study, ordered by “Number of records”. (DOC) [file pone.0105966.s003.doc]

**Appendix S3:** Reptiles captured in this study, ordered by “Number of records”**.** Multiple records under the same tile counted as 1.

| Common name | Species name | Size (mm) | Number of records | Number of plots | Number of tiles |
| --- | --- | --- | --- | --- | --- |
| Boulenger's skink | *Morethia boulengeri* | 100 | 261 | 57 | 141 |
| Delicate skink | *Lampropholis delicata* | 90 | 187 | 51 | 122 |
| Eastern three-toed earless skink | *Hemiergis talbingoensis.* | 100 | 66 | 20 | 37 |
| Common dwarf skink | *Menetia greyii* | 80 | 47 | 29 | 37 |
| Striped legless lizard | *Delma impar* | 200 | 26 | 11 | 21 |
| Grass skink | *Lamprophlois guitchenoti* | 90 | 19 | 14 | 17 |
| Eastern brown Snake | *Pseudonaja textilis* | 2000 | 11 | 8 | 10 |
| Olive legless lizard | *Delma inornata* | 400 | 10 | 7 | 10 |
| Common bearded dragon | *Pogona barbata* | 500 | 8 | 3 | 4 |
| Eastern striped skink | *Ctenotus robustus* | 300 | 5 | 3 | 3 |
| Shingleback lizard | *Trachydosaurus rugosus* | 400 | 4 | 1 | 2 |
| Copper-tailed skink | *Ctenotus taeniolatus* | 200 | 3 | 1 | 2 |
| Eastern blue-tongued lizard | *Tiliqua scincoides* | 500 | 2 | 2 | 2 |
| Spotted-backed skink | *ctenotus orientalis* | 300 | 2 | 2 | 2 |
| Eastern Three-lined Skink | *Bassiana duperreyi* | 200 | 1 | 1 | 1 |
| Jacky lizard | *Amphibolurus muricatus* | 400 | 1 | 1 | 1 |
| Pink-tailed worm lizard | *Aprasia parapulchella* | 180 | 1 | 1 | 1 |
| Four-fingered skink | *Carlia tetradactyla* | 150 | 1 | 1 | 1 |
| Red-throated skink | *Bassiana platynotum* | 180 | 1 | 1 | 1 |
| Legless lizards | *Delma. Sp* | 200-400 | 36 | 18 | 31 |
| unidentified skink |  | - | 89 | 45 | 80 |
