# Supplementary material for: Eaten Out of House and Home: Impacts of Grazing on Ground-Dwelling Reptiles in Australian Grasslands and Grassy Woodlands
Source: PLoS One. 2014 Dec 11;9(12):e105966. doi: 10.1371/journal.pone.0105966 (PMC4263405; doi:10.1371/journal.pone.0105966)
Supplement: Appendix S4 — Results of generalized linear mixed models for grass biomass, grass height and grass cover showing trends (Slope) including standard errors (SE). (DOC) [file pone.0105966.s004.doc]

**Appendix S4:** Results of generalized linear mixed models for grass biomass, grass height and grass cover showing trends (Slope) including standard errors (SE). Significance is indicated by the Wald statistic (χ^2^ ) and p-value as follows: *p<0.05, ** p<0.01, ***p<0.001. Non-significant trends are represented in grey.

| Response | Model term | d.f. | χ^2^ | Slope | SE | Graphical summary |
| --- | --- | --- | --- | --- | --- | --- |
| grass biomass | intercept |  |  | 582.60 | 21.40 |  |
|  | grass structure | 1 | 811.3*** | 391.50 | 13.70 | Appendix S5a |
| grass height | intercept |  |  | 7.38 | 0.13 |  |
|  | grass structure | 1 | 825.2*** | 2.40 | 0.08 | Appendix S5b |
| grass cover | intercept |  |  | 0.38 | 0.01 |  |
|  | grass structure | 1 | 299.6*** | 0.11 | 0.01 | Appendix S5c |
