# Supplementary material for: Eaten Out of House and Home: Impacts of Grazing on Ground-Dwelling Reptiles in Australian Grasslands and Grassy Woodlands
Source: PLoS One. 2014 Dec 11;9(12):e105966. doi: 10.1371/journal.pone.0105966 (PMC4263405; doi:10.1371/journal.pone.0105966)
Supplement: Appendix S5 — Graphical representation of generalized linear regression model summarized in Appendix S4. (DOC) [file pone.0105966.s005.doc]

**
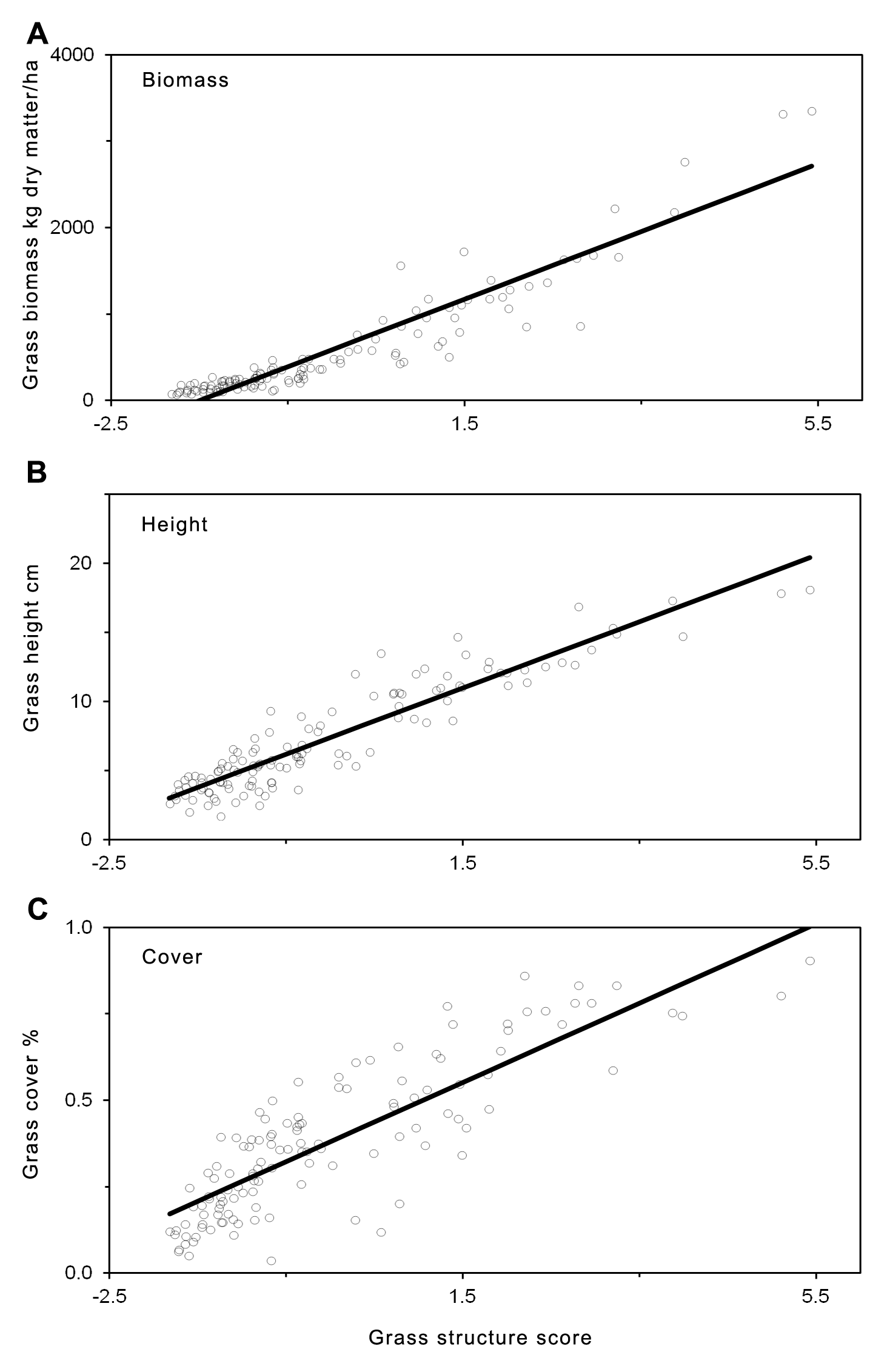
**

**Appendix S5:** Graphical representation of generalized linear regression model summarized in Appendix S4. The graphs show significant relationships between grass structure score a) grass biomass (kgDM/ha), b) grass height (cm), and c) grass cover (%). Fitted relationships are shown as a solid line, with actual values shown as open circles.
